# NUTRITION ASSESSMENT PROTOCOLS IN COMMUNITY-DWELLING OLDER ADULTS WITH CHRONIC WOUNDS

**DOI:** 10.1093/geroni/igz038.1441

**Published:** 2019-11-08

**Authors:** Rose Ann DiMaria-Ghalili, Sarah Charbonneau, Keyanna Bynum, Michael Neidrauer, Michael S Weingarten, Peter A Lewin

**Affiliations:** Drexel University, Philadelphia, Pennsylvania, United States

## Abstract

Older adults are at risk for altered nutritional status and functional impairment due to physiological (e.g., age-related changes, acute and chronic co-morbid conditions) and psychosocial factors (e.g., depression, loneliness, cognitive impairment). Those with alterations in nutritional and/or functional status are at risk for poor health outcomes-including delayed healing of chronic wounds. We will discuss our lessons learned when devising nutrition assessment protocols from our ongoing double-blind randomized control clinical trial testing the effectiveness of ultrasound treatment on healing chronic leg wounds. The discussion will focus on the following measures: the Mini-Nutritional Assessment, hand-grip strength assessment, and inflammatory biomarkers.

